# Supplementary material for: Large scale analyses of genotype-phenotype relationships of glycine decarboxylase mutations and neurological disease severity
Source: PLoS Comput Biol. 2020 May 18;16(5):e1007871. doi: 10.1371/journal.pcbi.1007871 (PMC7259800; doi:10.1371/journal.pcbi.1007871)
Supplement: S1 Fig — (PPTX) [file pcbi.1007871.s001.pptx]

## Slide 1
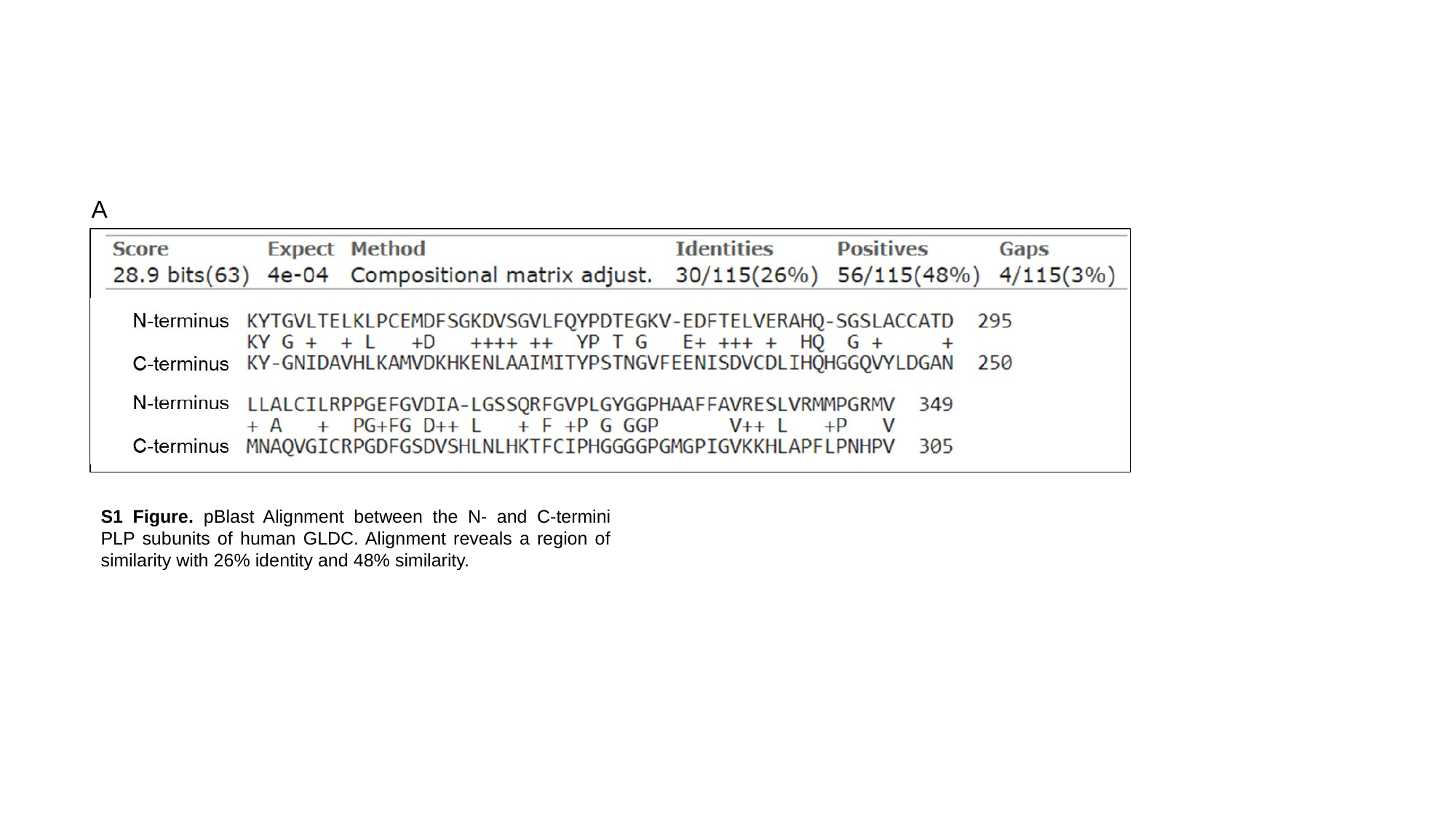

A
S1 Figure. pBlast Alignment between the N- and C-termini PLP subunits of human GLDC. Alignment reveals a region of similarity with 26% identity and 48% similarity.
