# Supplementary material for: Large scale analyses of genotype-phenotype relationships of glycine decarboxylase mutations and neurological disease severity
Source: PLoS Comput Biol. 2020 May 18;16(5):e1007871. doi: 10.1371/journal.pcbi.1007871 (PMC7259800; doi:10.1371/journal.pcbi.1007871)
Supplement: S2 Fig — (PPTX) [file pcbi.1007871.s002.pptx]

## Slide 1
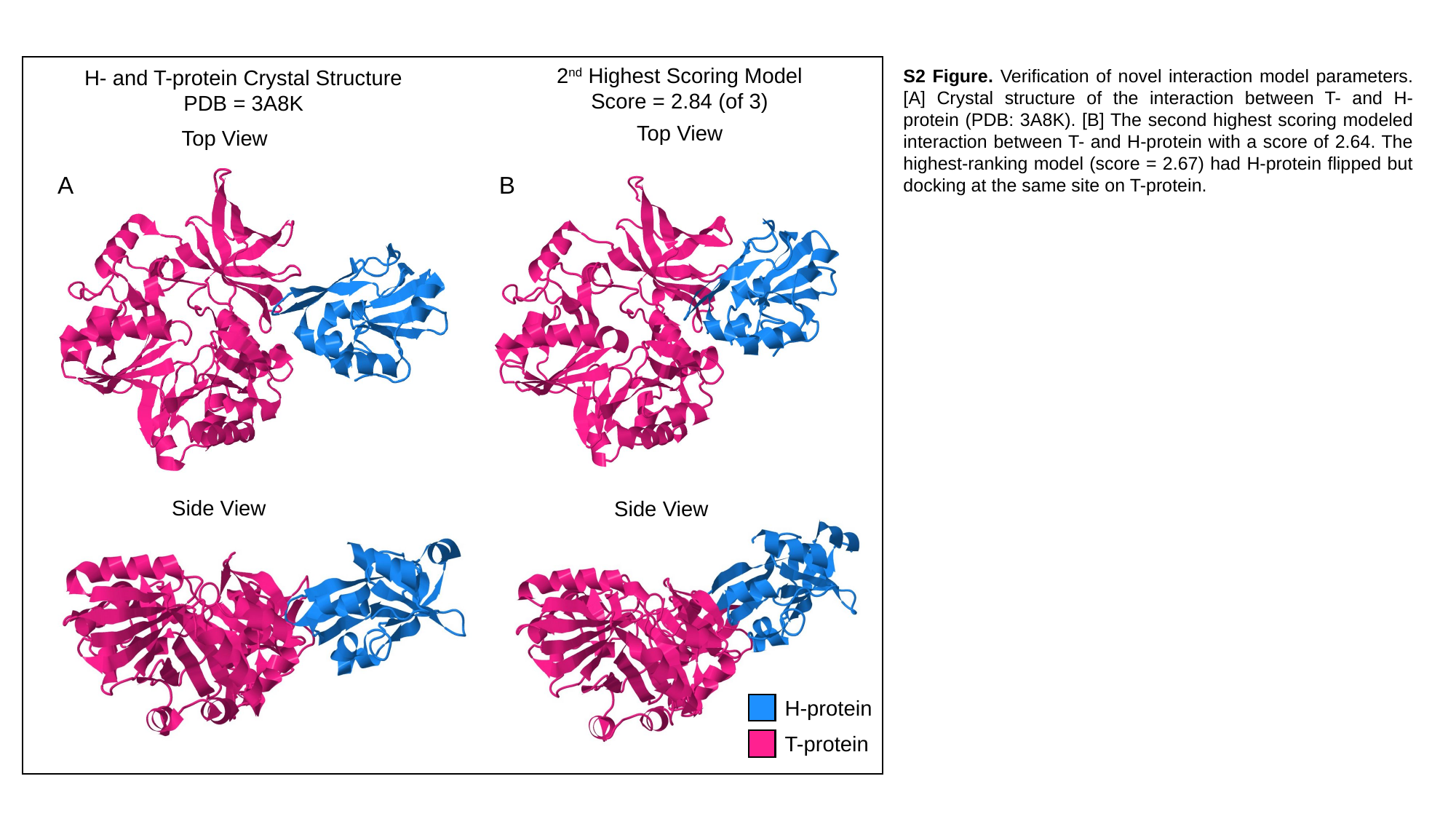

2nd Highest Scoring Model
Score = 2.84 (of 3)
S2 Figure. Verification of novel interaction model parameters. [A] Crystal structure of the interaction between T- and H-protein (PDB: 3A8K). [B] The second highest scoring modeled interaction between T- and H-protein with a score of 2.64. The highest-ranking model (score = 2.67) had H-protein flipped but docking at the same site on T-protein.
H- and T-protein Crystal Structure
PDB = 3A8K
Top View
Top View
A
B
Side View
Side View
H-protein
T-protein
