# Supplementary material for: Large scale analyses of genotype-phenotype relationships of glycine decarboxylase mutations and neurological disease severity
Source: PLoS Comput Biol. 2020 May 18;16(5):e1007871. doi: 10.1371/journal.pcbi.1007871 (PMC7259800; doi:10.1371/journal.pcbi.1007871)
Supplement: S3 Fig — (PPTX) [file pcbi.1007871.s003.pptx]

## Slide 1
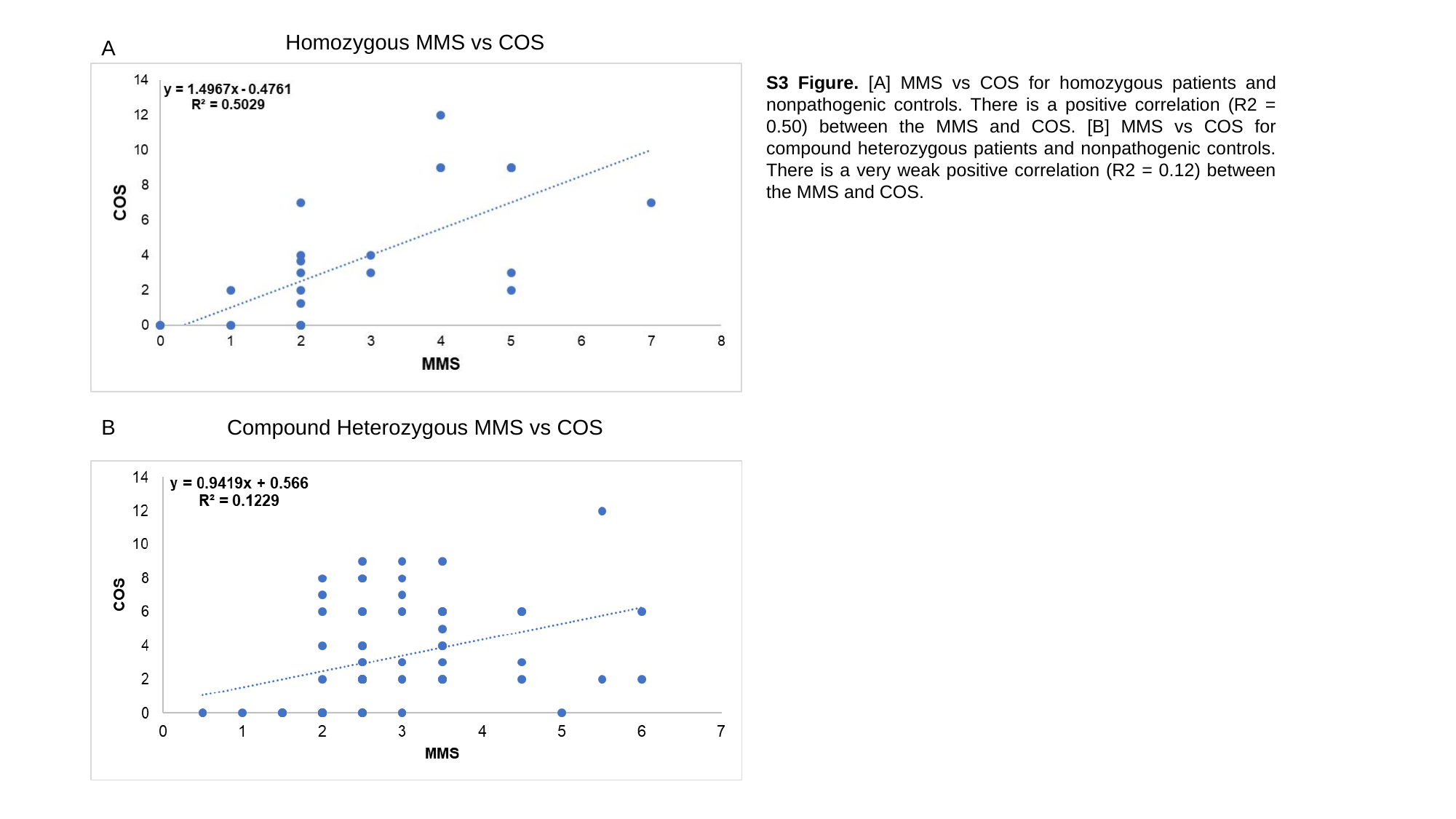

Homozygous MMS vs COS
A
S3 Figure. [A] MMS vs COS for homozygous patients and nonpathogenic controls. There is a positive correlation (R2 = 0.50) between the MMS and COS. [B] MMS vs COS for compound heterozygous patients and nonpathogenic controls. There is a very weak positive correlation (R2 = 0.12) between the MMS and COS.
B
Compound Heterozygous MMS vs COS
